# CeO_2_-Promoted PtSn/SiO_2_ as a High-Performance Catalyst for the Oxidative Dehydrogenation of Propane with Carbon Dioxide

**DOI:** 10.3390/nano12030417

**Published:** 2022-01-27

**Authors:** Li Wang, Guo-Qing Yang, Xing Ren, Zhong-Wen Liu

**Affiliations:** Key Laboratory of Syngas Conversion of Shaanxi Province, School of Chemistry & Chemical Engineering, Shaanxi Normal University, Xi’an 710119, China; kingli@snnu.edu.cn (L.W.); gqyang@snnu.edu.cn (G.-Q.Y.); rxqnmhhh@snnu.edu.cn (X.R.)

**Keywords:** oxidative dehydrogenation, propane, carbon dioxide, supported PtSn catalyst, ceria

## Abstract

The oxidative dehydrogenation of propane with CO_2_ (CO_2_-ODP) has been extensively investigated as a promising green technology for the efficient production of propylene, but the lack of a high-performance catalyst is still one of the main challenges for its industrial application. In this work, an efficient catalyst for CO_2_-ODP was developed by adding CeO_2_ to PtSn/SiO_2_ as a promoter via the simple impregnation method. Reaction results indicate that the addition of CeO_2_ significantly improved the catalytic activity and propylene selectivity of the PtSn/SiO_2_ catalyst, and the highest space-time yield of 1.75 g(C_3_H_6_)·g(catalyst)^−1^·h^−1^ was achieved over PtSn/SiO_2_ with a Ce loading of 6 wt%. The correlation of the reaction results with the characterization data reveals that the introduction of CeO_2_ into PtSn/SiO_2_ not only improved the Pt dispersion but also regulated the interaction between Pt and Sn species. Thus, the essential reason for the promotional effect of CeO_2_ on CO_2_-ODP performance was rationally ascribed to the enhanced adsorption of propane and CO_2_ originating from the rich oxygen defects of CeO_2_. These important understandings are applicable in further screening of promoters for the development of a high-performance Pt-based catalyst for CO_2_-ODP.

## 1. Introduction

Propylene is one of the most important raw materials for the chemical industry [1,2]. It is mainly produced by the steam cracking of naphtha and the byproduct of fluid catalytic cracking (FCC) of heavier oil fractions, which suffer from both low propylene yield and high energy consumption [3]. Moreover, these technologies cannot meet the continuously increased market demand for propylene. As a consequence and owing to the growing alternative supply of propane from the shale gas, the catalytically direct dehydrogenation of propane to propylene (PDH) has been attracting increased attention. However, the currently industrialized PDH process is still challenged by the quick deactivation of commercial PtSn/Al_2_O_3_ or CrO_x_/Al_2_O_3_ catalysts, low yields of propylene limited by thermodynamics, and high reaction temperatures [4,5]. To address these issues, the oxidative dehydrogenation of propane to propylene using O_2_, CO_2_, N_2_O, or SO_x_ as an oxidant is proposed as a more efficient route [6,7]. Among these alternative processes, the oxidative dehydrogenation of propane with greenhouse gas of CO_2_ (CO_2_-ODP) is the most attractive from an environmental viewpoint. On the one hand, in comparison with PDH, CO_2_-ODP can effectively enhance the equilibrium conversion of propane by removing the produced hydrogen [4,8]. On the other hand, a higher selectivity of propylene for CO_2_-ODP can be achieved in comparison with that achieved by using O_2_ as an oxidant, for which deep oxidation is an important issue. Moreover, the CO_2_-ODP process provides an attractively tandem approach via efficient production of propylene with simultaneous conversion of CO_2_ to CO [9,10]. Currently, a number of catalysts that predominantly contain supported metal oxides, e.g., Cr, V, In, Ga, and Zn, have been extensively investigated for CO_2_-ODP [4,11]. However, mechanistic understanding of the simultaneous activation of the C=O bonds in CO_2_ molecules and C-H bonds with the inhibited breaking of C-C bonds in propane molecules is very limited, which impedes the rational design of an efficient catalyst for CO_2_-ODP. Thus, a green CO_2_-ODP process is still far from industrial application. In fact, under industrially relevant reaction temperatures, C_3_H_8_ can react with CO_2_ via two distinct reaction routes, i.e., CO_2_-ODP (CO_2_ + C_3_H_8_ → C_3_H_6_ + CO + H_2_O) and the reforming of propane with CO_2_ (CO_2_-RP, 3CO_2_ + C_3_H_8_ → 6CO + 4H_2_) [12,13]. Moreover, the extents of the two reactions are kinetically determined by the employed catalyst. Generally, metal-oxide catalysts, such as VO_x_ and CrO_x_ are favorable for selectively breaking C-H bonds in propane to produce propylene, but they are less active for activation of CO_2_ molecules [14,15]. In contrast, the supported metals, such as Pt/CeO_2_ and Pd/CeZrAlO_x_, are good catalysts for the production of synthesis gas via the CO_2_-RP reaction, in which the selective splitting of C-C bonds in propane is favored, with the simultaneous activation of C=O bonds in CO_2_ [13,16]. Thus, as a result of the competing nature of the CO_2_-ODP and CO_2_-RP reactions, the favorable activation of C-H bonds with the inhibited breaking of C-C bonds in propane is one of the key requirements for development of a high-performance CO_2_-ODP catalyst.

In fact, contrary to Pt/CeO_2_, with a greater ability to break C-C bonds in alkane molecules, supported PtSn has already been applied as a commercial catalyst for the PDH reaction [17]. This rigorously indicates that the addition of SnO_2_ into Pt shifts the selective breaking of C-C bonds to the favorable activation of C-H bonds in propane molecules. Although the action nature of the added SnO_2_ on Pt is still not unambiguously revealed, the improved Pt dispersion and the electron transfer between Pt and Sn species are commonly agreed to be key factors in determining the activity and selectivity of propene for the PDH reaction [18,19]. This is further supported by quantitative studies on the interactions between Pt and Sn species, which can be tuned by adding promoters and changing the synthesis methods of the catalyst, including thermal treatment under different atmospheres [20].

In the case of CO_2_ activation, CeO_2_ with a high oxygen-storage capacity and abundant oxygen defects was extensively investigated as a support/promoter/catalyst for the reactions involving CO_2_ as a reactant [21]. In our previous study [22], CeO_2_ and CeO_2_-based solid solutions were found to be effective for converting CO_2_ into CO during the oxidative dehydrogenation of ethylbenzene with CO_2_, the ability of which is closely connected with oxygen defects over the oxides. Moreover, two functions of CeO_2_ as an additive over Pt-based catalysts for the PDH reaction were manifested as stabilizing Pt, suppressing coke deposition [23].

Based on these analyses, the combination of PtSn with CeO_2_ may create a good CO_2_-ODP catalyst, provided that the assisted activation of CO_2_ is achieved by oxygen defects over CeO_2_. Moreover, silica is widely used as a support for the CO_2_-ODP reaction [6], the side reactions of which are inhibited in comparison with Al_2_O_3_ with a higher acidity [24,25]. Thus, in this work, a high-performance CO_2_-ODP catalyst was developed by simply impregnating the CeO_2_ precursor into PtSn/SiO_2_. The PtSn/SiO_2_ catalyst, with a Ce loading of 6 wt%, showed a space-time yield of propene as high as 1.75 g(C_3_H_6_)·g(catalyst)^−1^·h^−1^ and superior stability, outperforming the state-of-the-art catalysts, including the supported oxides, such as CrO_x_, GaO_x_, and VO_x_, as well as the supported metals, such as Pd and Fe_3_Ni. The essence of the high CO_2_-ODP performance of CeO_2_-promoted PtSn/SiO_2_ is rigorously revealed as the enhanced adsorption of propane and CO_2_, which favors the selective breaking of C-H bonds in propane with the expedited tandem conversion of CO_2_ to CO.

## 2. Experimental

### 2.1. Catalyst Preparation

PtSnCe/SiO_2_, PtCe/SiO_2_, PtSn/SiO_2_, and SnCe/SiO_2_ catalysts were prepared by the sequential impregnation method, whereby the amounts of Pt, Sn, and Ce were fixed at 0.5, 0.9, and 6 wt%, respectively. The reagents of H_2_PtCl_6_·6H_2_O (Pt ≥ 37.5%, Shanghai Aladdin Biochemical Co., Ltd., Shanghai, China), SnCl_2_·6H_2_O (>99.9%, Guangdong Guanghua Sci-Tech Co., Ltd., Shantou, China), and Ce(NO_3_)_3_·6H_2_O (>99.0%, Shanghai Macklin Biochemical Co., Ltd., Shanghai, China) were chosen as the precursors of the Pt, Sn, and Ce species, respectively. The SiO_2_ support, with a specific BET surface area of 580 m^2^/g and an average pore diameter of 3.0 nm (Fuji Silysia Chemical Ltd., Kasugai Aichi, Japan), was pre-treated in air at 550 °C for 3 h. Impregnation was performed at 80 °C under stirring for 2 h, and the subsequent drying was carried out at 80 °C for 4 h. Following sequential impregnation with the desired amount of aqueous solution of H_2_PtCl_6_·6H_2_O, the acetone solution of SnCl_2_·2H_2_O and the aqueous solution of Ce(NO_3_)_3_·6H_2_O, PtSnCe/SiO_2_, PtCe/SiO_2_, PtSn/SiO_2_, and SnCe/SiO_2_ catalysts were obtained after calcining at 500 °C for 3 h under atmospheric conditions.

### 2.2. Catalyst Characterizations

N_2_ physical adsorption/desorption isotherms were measured on a Bel-sorp-Max instrument at −196 °C. Before each experiment, the sample was degassed at 300 °C under vacuum for 10 h. Specific surface area and pore-size distribution (PSD) were calculated by the Brunauer–Emmett–Teller equation (BET) and Barrett–Joyner–Halenda method (BJH), respectively.

X-ray diffraction (XRD) patterns were recorded on an X-ray diffractometer (Bruker D8 Advance) equipped with Cu-Kα radiation (40 kV, 40 mA). The sample was scanned from the 2θ of 10 to 80° with a rate of 0.2 s/step.

Transmission electron microscopy (TEM) images were obtained with a high-resolution transmission electron microscope (Tecnai G2 F20, FEI) operated at 200 kV. Before measurement, the fresh sample was pre-reduced at 500 °C in 10 vol% H_2_/Ar for 1 h. Then, about 2 mg of the reduced sample was ultrasonically dispersed in anhydrous ethanol (2 mL). After 1 h, two drops of the suspension were deposited on a carbon-enhanced copper grid and dried at 60 °C in air for 0.5 h.

H_2_-O_2_ titration experiments were carried out on a Micromeritics Autochem 2920 instrument to determine the dispersion of Pt. For each test, a total of 150 mg of the sample was pre-reduced at 500 °C for 1 h in 10 vol% H_2_/Ar (30 mL/min). After cooling to 50 °C in Ar, a flow of 3 vol% O_2_/Ar (30 mL/min) was pulsed until the consumption peaks became stable. Subsequently, the sample was purged under an Ar flow for 1 h, and consecutive pulses of 10 vol% H_2_/Ar (0.50 mL) were performed. By assuming that the adsorption stoichiometry factor of Pt/H_2_ equals to 2/3, according to references [26,27], the dispersion of Pt was calculated using the following Equation (1).

(1) $$\mathrm{Dispersion}\;\left(\%\right)=\frac{V_{H_{2}}{\times \;f\;\times \;M}_{\mathrm{Pt}}}{W_{\mathrm{Pt}}\;\times \;22,414}\times \;100$$

where $V_{H_{2}}$ is the volume of adsorbed H_2_ (mL), f is the stoichiometry factor, M_Pt_ is the atomic weight of Pt (g/mol), and W_Pt_ is the weight of the supported Pt on the sample (g).

Experiments concerning temperature-programmed reduction of H_2_ (H_2_-TPR) were carried out on a Micromeritics Autochem 2920 instrument. About 100 mg of the sample was pre-treated at 350 °C for 0.5 h under an Ar stream. After cooling to 50 °C, H_2_-TPR was performed from 50 to 800 °C at a heating rate of 10 °C/min under a 10 vol% H_2_/Ar flow (30 mL/min). H_2_ consumption was monitored and determined by a pre-calibrated thermal conductivity detector (TCD).

X-ray photoelectron spectroscopy (XPS) was conducted on an X-ray photoelectron spectrometer (KRATOS Analytical Ltd., Manchester, UK) equipped with an Al-Kα radiation source (1486.6 eV). Before measurements, all the samples were pre-reduced at 500 °C for 1 h in 10 vol% H_2_/Ar. The C 1s spectrum at 284.6 eV was applied to calibrate the binding energy.

Diffuse reflectance infrared Fourier transform spectroscopy (DRIFTS) of adsorbed CO (CO-DRIFTS) was carried out on a Nicolet iS50 instrument (Thermo Scientific) equipped with an in situ cell. Firstly, the sample was reduced in situ at 500 °C for 1 h in 10 vol% H_2_/Ar with a flow rate of 30 mL/min. After this, the sample was cooled to 30 °C and purged with Ar. Then, 10 vol% CO/Ar with a flow rate of 30 mL/min was introduced in the cell for 0.5 h. Afterwards, the sample was purged with Ar to remove any physically adsorbed CO on the surface of sample, and DRIFTS spectra were recorded. Prior to each experiment, the background spectra were recorded.

Experiments concerning temperature-programmed desorption of C_3_H_8_/CO_2_/C_3_H_6_ (C_3_H_8_/CO_2_/C_3_H_6_-TPD) were performed on a Micromeritics Autochem 2920 instrument. About 100 mg of the sample was pre-reduced at 500 °C for 1 h in 10 vol% H_2_/Ar. After this, the sample was cooled to 70 °C and purged with Ar. Then, the pre-treated sample was saturated with pure C_3_H_8_, CO_2_, or C_3_H_6_ with a flow rate of 30 mL/min for 1 h. Afterwards, the sample was purged by an Ar stream for 1 h, and temperature-programmed desorption of C_3_H_8_/CO_2_/C_3_H_6_ was performed from 70 to 600 °C at a heating rate of 10 °C/min, respectively. The amount of desorbed C_3_H_8_/CO_2_/C_3_H_6_ was monitored and determined by a pre-calibrated thermal conductivity detector (TCD).

Thermogravimetric and differential scanning calorimetry analyses (TG-DSC) of the spent catalysts were carried out on a Q600SDT Thermoanalyzer System (TA Instruments). For each test, about 5 mg of the spent catalyst was heated from 50 to 800 °C with a heating ramp of 10 °C/min^−1^ in a flow of air.

Raman spectra were obtained on a confocal microprobe laser Raman spectrometer (HORIBA Jobin Yvon) with an excitation laser beam of 532 nm. Spectra in the range of 1000–2000 cm^−1^ were recorded at room temperature to study the type of deposited coke over the spent catalysts.

### 2.3. Catalytic Tests

Catalytic tests for CO_2_-ODP were carried out in a quartz fixed-bed reactor (6 mm, i.d.) under 550 °C and atmospheric pressure. For each test, 0.25 g of the catalyst (40–60 mesh) diluted with 0.5 g of quartz sand (40–60 mesh) was loaded into the reactor. Firstly, the catalyst was pre-reduced with 20 vol% H_2_/He at 500 °C for 1h. After that, the reactor was heated to 550 °C in a He flow, and the gas mixture of Ar/C_3_H_8_/CO_2_/He in a molar ratio of 1/4/4/16 with a total flow rate of 50 mL/min was introduced into the reactor. The products were analyzed by an online gas chromatograph (GC7920, Peking CEAULIGHT) equipped with FID (Porapak Q column) and TCD (TDX-01) detectors. By using Ar as an internal standard, propane and CO_2_ conversion, selectivity of different gas products (CH_4_, C_2_H_4_, C_2_H_6_, and C_3_H_6_), and propylene yield were calculated by Equations (2)–(5).

(2) $$C_{3}H_{8}\;\mathrm{conversion}=\frac{{\left[F_{C_{3}H_{8}}\right]}_{\mathrm{inlet}}-{\left[F_{C_{3}H_{8}}\right]}_{\mathrm{outlet}}}{{\left[F_{C_{3}H_{8}}\right]}_{\mathrm{inlet}}}\;\times \;100\%$$

(3) $${\mathrm{CO}}_{2}\;\mathrm{conversion}=\frac{{\left[F_{{\mathrm{CO}}_{2}}\right]}_{\mathrm{inlet}}-{\left[F_{{\mathrm{CO}}_{2}}\right]}_{\mathrm{outlet}}}{{\left[F_{{\mathrm{CO}}_{2}}\right]}_{\mathrm{inlet}}}\;\times \;100\%$$

(4) $$\mathrm{Selectivity}\;\mathrm{of}\;\mathrm{products}\;i=\frac{n_{i}\times {\left[F_{i}\right]}_{\mathrm{outlet}}}{3\times \left({\left[F_{C_{3}H_{8}}\right]}_{\mathrm{inlet}}-{\left[F_{C_{3}H_{8}}\right]}_{\mathrm{outlet}}\right)}\;\times \;100\%$$

(5) $$C_{3}H_{6}\;\mathrm{yield}=\frac{{\left[F_{C_{3}H_{6}}\right]}_{\mathrm{outlet}}}{{\left[F_{C_{3}H_{8}}\right]}_{\mathrm{inlet}}}\;\times \;100\%$$

The carbon balances calculated from Equation (6) were very close to 100% (98.5 ± 1.5%) for all of the experiments (Figure S1), and thus, the C_3_H_8_-based selectivity of CO, i.e., CO produced from C_3_H_8_, was calculated by subtracting the sum of the hydrocarbon products (CH_4_ + C_2_H_4_ + C_2_H_6_ + C_3_H_6_) from 100% with Equation (7).

(6) $$\mathrm{Carbon}\;\mathrm{balance}=\frac{\sum_{i}n_{i}\times {\left[F_{i}\right]}_{\mathrm{outlet}}{+3\;\times \;[F}_{C_{3}H_{8}}{]}_{\mathrm{outlet}}{+[F}_{\mathrm{CO}}{]}_{\mathrm{outlet}}{+[F}_{{\mathrm{CO}}_{2}}{]}_{\mathrm{outlet}}}{{3\;\times \;[F}_{C_{3}H_{8}}{]}_{\mathrm{inlet}}{+[F}_{{\mathrm{CO}}_{2}}{]}_{\mathrm{inlet}}}\;\times \;100\%$$

(7) $$\mathrm{CO}\;\mathrm{selectivity}\;=\left(1-\sum_{i}\frac{n_{i}\times {\left[F_{i}\right]}_{\mathrm{outlet}}}{3\times \left({\left[F_{C_{3}H_{8}}\right]}_{\mathrm{inlet}}-{\left[F_{C_{3}H_{8}}\right]}_{\mathrm{outlet}}\right)}\right)\times 100\%$$

where, $F_{C_{3}H_{8}}$, $F_{{\mathrm{CO}}_{2}}$, and $F_{\mathrm{CO}}$ are the volumetric flow rate (mL/min) of C_3_H_8_, CO_2_, and CO, respectively; i stands for the detected hydrocarbon product, i.e., CH_4_, C_2_H_6_, C_2_H_4_, and C_3_H_6_; and F_i_ and n_i_ represent the flow rate and carbon number of the hydrocarbon product, respectively.

## 3. Results

### 3.1. Catalytic Results of CO_2_-ODP

**Figure 1:**
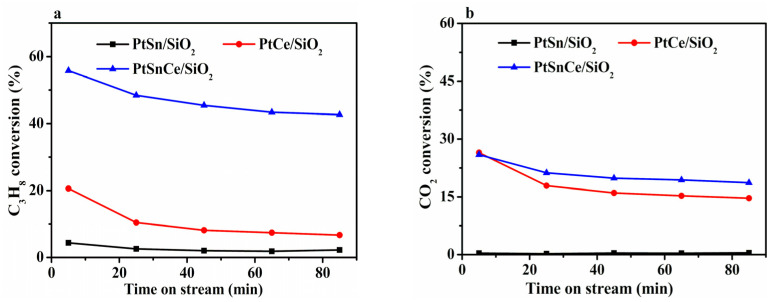

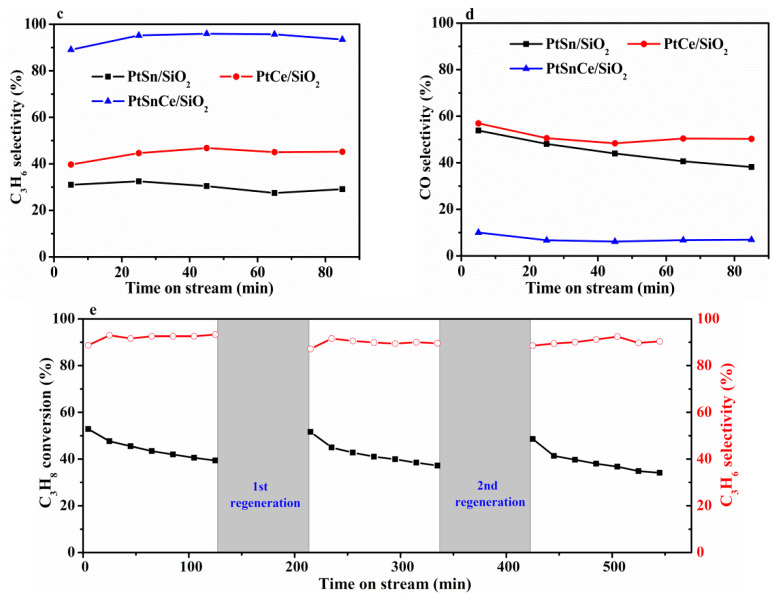
Time-on-stream conversions of C_3_H_8_ (**a**) and CO_2_ (**b**); C_3_H_6_ selectivity (**c**) and CO selectivity (**d**) for CO_2_-ODP catalyzed by PtSn/SiO_2_, PtCe/SiO_2_, and PtSnCe/SiO_2_; and time-on-stream C_3_H_8_ conversions/C_3_H_6_ selectivity over the regenerated PtSnCe/SiO_2_ (**e**) under the conditions of T = 550 °C, Ar/C_3_H_8_/CO_2_/He molar ratio = 1/4/4/16, total flow rate = 50 mL/min, and GHSV = 12,000 mL·g^−1^·h^−1^ (conditions for the regeneration: T = 500 °C, air flow = 30 mL/min, t = 30 min).

The time-on-stream (TOS) results of CO_2_-ODP over PtSn/SiO_2_, PtCe/SiO_2_, and PtSnCe/SiO_2_ are shown in Figure 1. Indeed, PtSn/SiO_2_ showed a very low propane conversion of 4.4% at a TOS of 5 min (Figure 1a). In contrast, a significantly higher propane conversion was achieved over the Ce-containing catalysts, and PtSnCe/SiO_2_ showed the highest initial propane conversion of 55.8%. Taking the activity as the propane conversion at a TOS of 5 min, an increased order of PtSn/SiO_2_ < PtCe/SiO_2_ << PtSnCe/SiO_2_ was observed. Moreover, the blank experimental results of CO_2_-ODP indicate a propane conversion of less than 2.5% over SnCe/SiO_2_ (Figure S2). Thus, the Pt species over the catalysts are responsible for converting propane, the activity of which is associated with the added SnO_2_ and/or CeO_2_. Where the initial activity indexed by the CO_2_ conversion at a TOS of 5 min (Figure 1b) was concerned, PtSn/SiO_2_ showed negligible activity. In contrast, a significantly high initial CO_2_ conversion of 26.4% over PtCe/SiO_2_ and 25.9% over PtSnCe/SiO_2_ was achieved. In the case of stability, Figure 1a,b clearly shows that PtSnCe/SiO_2_ was the most stabile catalyst at a TOS of 80 min, leading to the concurrently decreased conversions of propane and CO_2_ in the same order of PtSnCe/SiO_2_ > PtCe/SiO_2_ >> PtSn/SiO_2_ with increasing TOS.

Concerning product distribution, propylene selectivity was varied to a relatively large extent over these catalysts (Figure 1c). In the case of PtSn/SiO_2_, the lowest propylene selectivity of 30.9% was observed at a TOS of 5 min. On the contrary, a higher propylene selectivity was achieved over the Ce-containing catalysts, and the highest initial propylene selectivity of 89.1% was reached over PtSnCe/SiO_2_. With increasing TOS, propylene selectivity at the end of the reaction was decreased in the order of PtSnCe/SiO_2_ (93.4%) >> PtCe/SiO_2_ (45.0%) > PtSn/SiO_2_ (27.4%). To understand the side reactions, the selectivity of gaseous byproducts originating from C_3_H_8_ were examined, and the results are shown in Figure 1d and Figure S3. In the case of PtSnCe/SiO_2_ and PtCe/SiO_2_, CO was the predominant byproduct, while the selectivity of C_2_H_6_, C_2_H_4_, and CH_4_ was very low. This indicates that CO_2_-RP may be the main side reaction over these catalysts in comparison with cracking [13,28]. The significantly higher CO selectivity of 56.9% at a TOS of 5 min was observed over PtCe/SiO_2_, indicating the more favorable breaking of C-C bonds in propane. In contrast, PtSnCe/SiO_2_ showed a very low CO selectivity of about 10%, coinciding with the significantly high propylene selectivity. Concerning PtSn/SiO_2_, although the calculated selectivity of CO was 53.9% at a TOS of 5 min, supporting the occurrence of CO_2_-RP, its error may be large due to the very low CO_2_ conversion (Figure 1b). Thus, the CO selectivity of PtSn/SiO_2_ is not further discussed with that of PtSnCe/SiO_2_ and PtCe/SiO_2_.

To show the superior performance of the PtSnCe/SiO_2_ catalyst for CO_2_-ODP, the calculated space-time yield of propylene (STY_C3H6_) over PtSnCe/SiO_2_ and those over different types of catalysts with the best performance from the representative literature are summarized in Table S1. Among the reported catalysts, including CrO_x_, VO_x_, GaO_x_, Pd, and Pt, the highest STY_C3H6_ of 0.63 g(C_3_H_6_)·g(catalyst)^−1^·h^−1^ was observed at a reaction temperature of 600 °C over CrO_x_-doped mesoporous silica spheres (7.07Cr/MSS-2). In our case, however, the PtSnCe/SiO_2_ catalyst showed an initial STY_C3H6_ as high as 1.75 g(C_3_H_6_)·g(catalyst)^−1^·h^−1^ at a reaction temperature of 550 °C, which is significantly higher than those over the reported catalysts. Moreover, 1.16 g(C_3_H_6_)·g(catalyst)^−1^·h^−1^ was still achieved, even at a TOS of 6 h, indicating a superior catalytic stability. The durability of PtSnCe/SiO_2_ was further investigated by the reaction/regeneration cycles, the regeneration of which is performed at 500 °C in an air flow for 30 min. As shown in Figure 1e, the CO_2_-ODP performance of the catalyst regenerated for two times was very similar to that of the fresh catalyst, indicating the good durability of PtSnCe/SiO_2_. Thus, the catalytic results clearly reflect the superiority of the PtSnCe/SiO_2_ catalyst for CO_2_-ODP.

### 3.2. Textural and Structural Properties

The N_2_ adsorption-desorption isotherms of PtSn/SiO_2_, PtCe/SiO_2_, and PtSnCe/SiO_2_ are shown in Figure S4a. According to the IUPAC classification, all of the catalysts exhibited a similar type-IV isotherm, indicating the presence of mesopores. Moreover, the appearance of an H1-type hysteresis loop over these catalysts occurred at p/p_0_ = 0.4–0.6 characterizing the uniform spherical pores. These observations were more directly reflected from the PSD patterns determined by the BJH method. As indicated by Figure S4b, a very narrow and sharp PSD peak at about 3 nm was observed for all of these catalysts. From the calculated textural parameters summarized in Table 1, the BET specific surface area was slightly decreased from 568.9 to 527.3 m^2^/g in the order of PtSn/SiO_2_ > PtCe/SiO_2_ > PtSnCe/SiO_2_. In the cases of mean pore size and total pore volume, the changes were also very limited. These results suggest very similar textural properties of the samples due to the minimal loadings of Pt, Sn, and/or Ce species over the same silica support.

**Figure 2:**
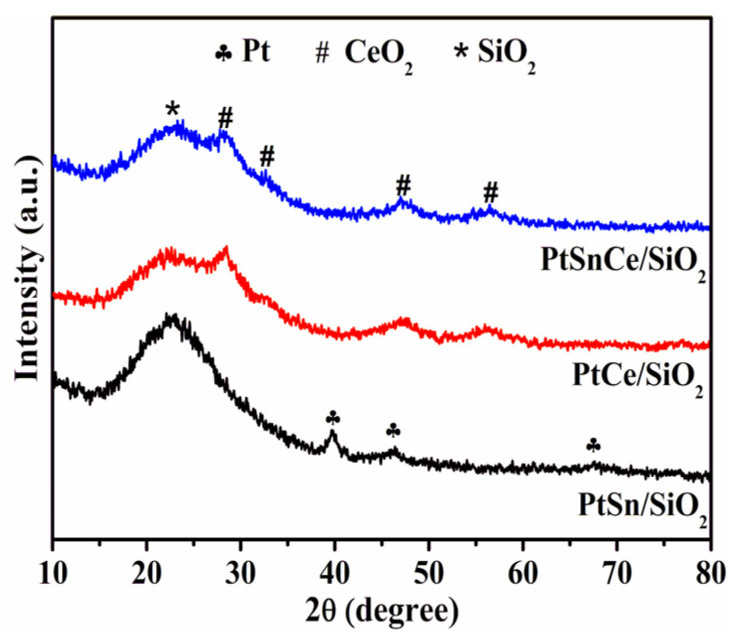
XRD patterns of the PtSn/SiO_2_, PtCe/SiO_2_, and PtSnCe/SiO_2_ catalysts.

XRD patterns of the catalysts are shown in Figure 2. All of the catalysts exhibited a broad XRD peak at ~22.6°, corresponding to the amorphous nature of the SiO_2_ support [29]. In the case of PtSn/SiO_2_, the characteristic diffractions at 2θ of 39.8, 46.2, and 67.4° were clearly observed, which were assigned to (111), (200), and (220) crystal planes of the cubic Pt metal, respectively [30,31]. In contrast, when CeO_2_ was present, the XRD peaks ascribed to Pt metal disappeared, and only the characteristic diffractions at 2θ of 28.5, 33.1, 47.5, and 56.3° conclusively attributed to the (111), (200), (220), and (311) crystal planes of the cubic fluorite structure of CeO_2_, respectively [22,32], were clearly observed over PtCe/SiO_2_ and PtSnCe/SiO_2_. This indicates that the addition of Ce can significantly improve the dispersion of Pt metal, and the SnO_2_ species is present as the amorphous or highly dispersed nature.

**Figure 3:**
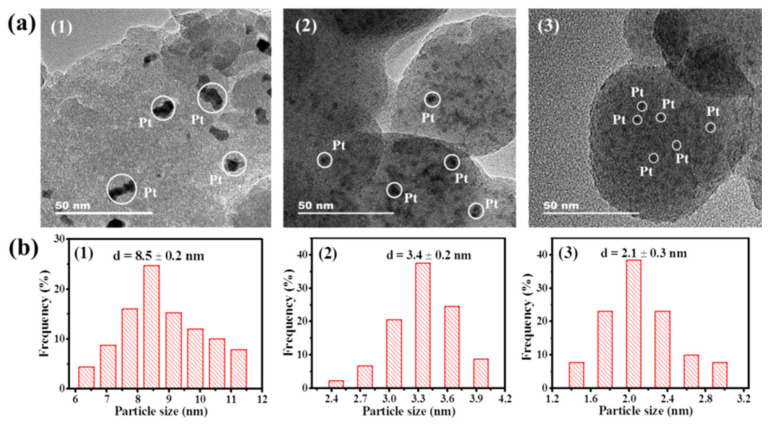
TEM images (**a**) and size distributions of Pt particles (**b**) for the reduced catalysts of PtSn/SiO_2_ (**1**), PtCe/SiO_2_ (**2**), and PtSnCe/SiO_2_ (**3**).

To directly observe the Pt particles, the catalysts pre-reduced in 10 vol% H_2_/Ar at 500 °C for 1 h were investigated by TEM analysis. As shown in Figure 3a, Pt particles were clearly observed over all of the catalysts. PtSn/SiO_2_ showed the largest Pt particles, while the significantly smaller Pt particles were present over PtCe/SiO_2_ and PtSnCe/SiO_2_, which supports the XRD results. To make a quantitative comparison, statistics analysis was performed; the Pt particle-size distribution histograms are given in Figure 3b, and the average diameter of Pt is summarized in Table 1. The metallic Pt size was continuously decreased from 8.5 ± 0.2 to 2.1 ± 0.3 nm in the order of PtSn/SiO_2_ >> PtCe/SiO_2_ > PtSnCe/SiO_2_, indicating that the addition of CeO_2_ can significantly improve the dispersion of Pt metal. This is further supported by the H_2_-O_2_ titration results, in which the calculated Pt dispersion was continuously increased from 13.4 to 41.3% in the order of PtSn/SiO_2_ < PtCe/SiO_2_ < PtSnCe/SiO_2_ (Table 1). Generally, the addition of SnO_2_ can effectively improve the Pt dispersion. However, this is contradictory to the observations over PtSn/SiO_2_, in which a Pt size as high as 8.5 ± 0.2 nm was observed. As for the reason, it has been reported that PtSn/SiO_2_ catalysts directly calcined in an oxidative atmosphere during the preparation process show poor Pt dispersion [33] due to the weak interaction between Pt and SnO_2_ on the SiO_2_ support. This is in agreement with our experimental results, in which all of the catalysts were obtained by calcining in air at 500 °C after impregnation, as described in Section 2.1. In contrast, when CeO_2_ was introduced, the Pt size was significantly decreased over PtCe/SiO_2_ and PtSnCe/SiO_2_. Taking these results into account, the lower Pt size of 2.1 nm for PtSnCe/SiO_2_, compared to that of PtCe/SiO_2_, indicates that in addition to increasing the Pt dispersion, the presence of CeO_2_ can improve the interaction between Pt and SnO_2_, leading to the highest Pt dispersion over PtSnCe/SiO_2_.

### 3.3. Reduction Behavior

**Figure 4:**
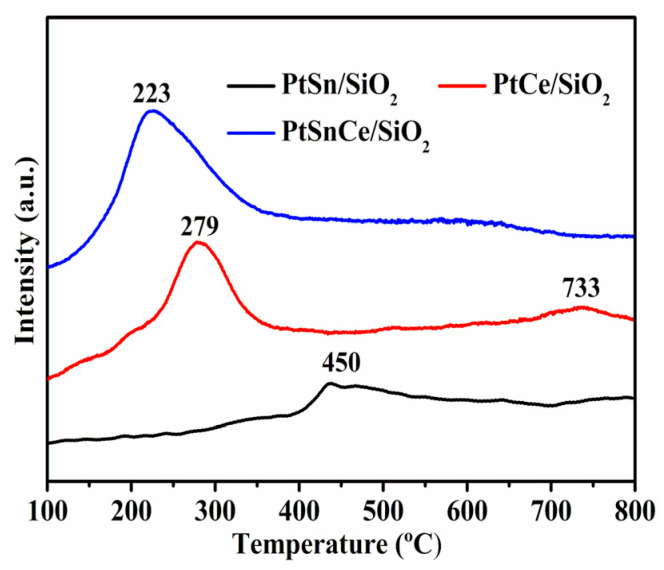
H_2_-TPR profiles of the PtSn/SiO_2_, PtCe/SiO_2_, and PtSnCe/SiO_2_ catalysts.

The redox properties of the catalysts were analyzed by H_2_-TPR. As shown in Figure 4, for the PtSn/SiO_2_ catalyst, a weak and broad reduction peak was observed at ~450 °C, which can be can be attributed to the reduction of SnO_2_ [34,35]. In the case of PtCe/SiO_2_, two reduction peaks were observed at 279 °C and 733 °C. The first peak at 279 °C was attributed to the reduction of active oxygen species over the CeO_2_ surface, while the very weak peak at 733 °C corresponded to the reduction of the lattice oxygen over the bulk CeO_2_ [22,36]. Concerning PtSnCe/SiO_2_, only one reduction peak was observed at 233 °C, attributed to the reduction of surface oxygen species. The reduction-peak temperature below 600 °C was continuously decreased in the order of PtSn/SiO_2_ >> PtCe/SiO_2_ > PtSnCe/SiO_2_, while the amount of the H_2_ consumption calculated from the reduction peak during H_2_-TPR below 600 °C (Table 2) was increased in the order of PtSn/SiO_2_ << PtCe/SiO_2_ < PtSnCe/SiO_2_, leading to the greatest reducibility of PtSnCe/SiO_2_. It has been reported that the hydrogen spillover effect induced from the interaction between the Pt and SnO_2_/CeO_2_ can accelerate the reduction of oxygen over the catalyst, and a higher dispersion of Pt commonly leads to a greater reducibility [37,38]. Based on this explanation, the H_2_-TPR results were well understood when the dispersion of Pt, as discussed in Section 3.2, was taken into account. As a result of the highest Pt dispersion over PtSnCe/SiO_2_, the reduction of oxides over the catalysts was enhanced due to the strongest hydrogen spillover effect, leading to the lowest reduction-peak temperature and the greatest amount of H_2_ consumption.

### 3.4. Chemical States

XPS analysis was used to study the surface chemical states of the catalysts. Before measurements, all of the catalysts were pre-reduced in 10 vol% H_2_/Ar at 500 °C for 1 h. Ce 3d spectra are usually fitted with eight Gaussian-Lorentzian peaks corresponding to two pairs of spin-orbit doubles [32,39]. As shown in Figure S5, the peaks labeled as v, v″, v‴ and u, u″ u‴ were assigned to the ionization of Ce^4+^ 3d_5/2_ and Ce^4+^ 3d_3/2_, respectively, while the peaks marked with v′ and u′ were originated from Ce^3+^ 3d_5/2_ and Ce^3+^ 3d_3/2_, respectively. Based on those peak areas, the relative content of Ce^3+^ was calculated, defined as the ratio of Ce^3+^/(Ce^3+^ + Ce^4+^). As shown in Table 2, the relative content of Ce^3+^ over PtSnCe/SiO_2_ is clearly higher than that of PtCe/SiO_2_, indicating the formation of more oxygen defects after the reduction. This is consistent with the significantly greater amount of H_2_ consumption during the H_2_-TPR over the fresh PtSnCe/SiO_2_ than that over PtCe/SiO_2_ (Table 2), resulting from the stronger hydrogen spillover effect due to the greater dispersion of Pt (Table 1).

**Figure 5:**
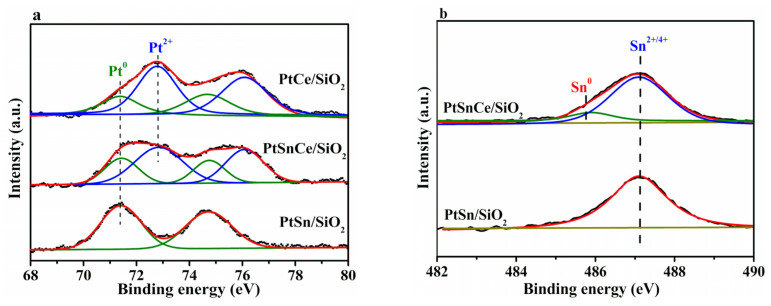
Pt 4f (**a**) and Sn 3d (**b**) XPS spectra of the reduced catalysts.

As show in Figure 5a, the binding energies at around 71.4 eV for 4f_7/2_ and 74.7 eV for 4f_5/2_ were clearly observed over PtSn/SiO_2_, indicating the presence of metallic Pt^0^ [40]. In contrast, when CeO_2_ was added, the binding energies of Pt 4f were clearly increased, and the XPS peaks at 72.8 eV for 4f_7/2_ and 76.1 eV for 4f_5/2_ assigned to Pt^2+^ species [40] were clearly observed over PtCe/SiO_2_ and PtSnCe/SiO_2_. Following the results of deconvolution of the Pt 4f peaks (Figure 5a), the relative content of Pt^0^ and Pt^2+^ was calculated by respective peak area. As shown in Table 2, PtSn/SiO_2_ showed the exclusively metallic Pt species, which is consistent with XRD results (Figure 2). Contrarily, the relative content of Pt^2+^ was as high as 69.5% and 66.9% for PtCe/SiO_2_ and PtSnCe/SiO_2_, respectively. According to references [41,42], the presence of Pt^2+^ over the CeO_2_-containing catalysts originates from the strong interaction between Pt and CeO_2_, which may be the key reason for the greater dispersion of Pt over PtCe/SiO_2_ and PtSnCe/SiO_2_ than over PtSn/SiO_2_. This is supported by the TEM and H_2_-O_2_ titration results (Figure 3 and Table 1). As shown in Figure 5b, the Sn 3d_5/2_ at about 487.0 eV was deconvoluted to analyze the chemical state of Sn. In the case of PtSn/SiO_2_, a symmetric Sn 3d_5/2_ XPS peak with a binding energy of 487.2 eV was observed, indicating the presence of only SnO_x_ species, as reported in [28]. However, besides oxide species, a small amount of Sn^0^ species located at 485.7 eV was observed over PtSnCe/SiO_2_. As for the reason, it is noteworthy that PtSnCe/SiO_2_ showed a slightly higher content of Pt^0^ than PtCe/SiO_2_ (Table 2), which may originate from the improved interaction between SnO_2_ and Pt due to the presence of CeO_2_. This coincides well with the presence of Sn^0^ species, indicating the possible formation of Pt-Sn bimetallic nanoparticles [28,43].

### 3.5. CO-DRIFTS Studies

**Figure 6:**
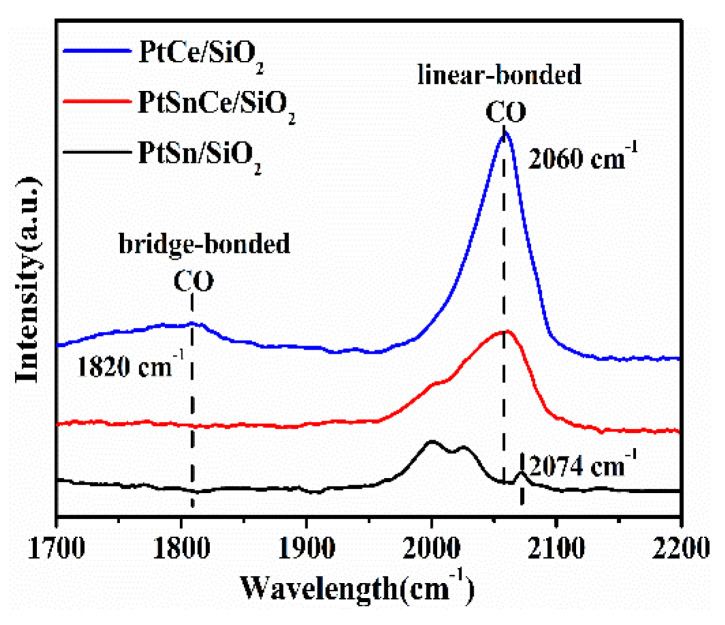
DRIFT spectra of CO adsorption on the reduced catalysts.

To further investigate the structural and electronic properties of Pt, DRIFTS experiments were performed by using CO as a probing molecule since its adsorption on Pt surfaces has been well studied. As shown in Figure 6, two overlapping bands were clearly observed in the case of PtSn/SiO_2_ at 2000 and 2024 cm^−1^, assigned to Si-H stretching vibrations in the different SiO_2_ configuration [44]. Moreover, a very weak peak was detected at about 2074 cm^−1^, assigned to the linearly bonded CO on Pt^0^ terraces, indicating the presence of large, highly coordinated nanoparticles [45,46]. When PtCe/SiO_2_ and PtSnCe/SiO_2_ were considered, significantly weakened and even disappeared peaks were observed for the Si-H stretching vibrations, which may be due to the coverage of CeO_2_ on the SiO_2_ surface. Moreover, strong band was observed at ~2060 cm^−1^, ascribed to the linearly bonded CO on Pt^0^, with intermediate coordination sites, such as edges or steps sites [47], indicating the high dispersion of Pt over these two Ce-containing catalysts [48,49]. This is supported by the results of TEM and H_2_-O_2_ titration (Figure 3 and Table 1). Noteworthy, besides the band at 2060 cm^−1^, a weak adsorption band was detected over PtCe/SiO_2_ at 1820 cm^−1^, ascribed to the bridge-bonded CO on two neighboring Pt atoms [24]. However, it disappeared in the case of PtSnCe/SiO_2_, accompanying a decreased intensity of the linear adsorption peak at 2060 cm^−1^. The disappeared bridge-bonded CO over PtSnCe/SiO_2_ suggests that the SnO_2_ breaks the ensemble of Pt atoms and forms a checkerboard Pt-Sn surface structure [24,50] because CO does not adsorb at the bridge sites between Sn and Pt. The decreased intensity of the peak at 2060 cm^−1^, in comparison with PtCe/SiO_2_, can be explained as the reduced surface coverage of CO due to the presence of SnO_2_ [51]. These results indicate that the presence of CeO_2_ on a PtSn/SiO_2_ catalyst can not only improve the Pt dispersion but also improve the interaction between Pt and SnO_2_.

## 4. Discussion

### 4.1. Key Factors of Catalytic Activity

**Figure 7:**
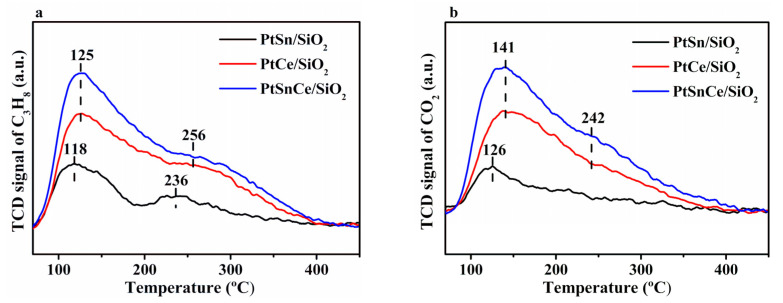
C_3_H_8_ (**a**) and CO_2_-TPD (**b**) profiles over the reduced catalysts.

As indicated by the results in Section 3.1 and Section 3.2, the initial C_3_H_8_ conversion at a TOS of 5 min was increased in the order of PtSnCe/SiO_2_ >> PtCe/SiO_2_ >> PtSn/SiO_2_, coinciding well with the dispersion of Pt over the catalysts. This indicates that the amount of active Pt species is the key factor determining the activation of propane in the course of CO_2_-ODP, which is consistent with the reported results for PDH [52]. When the activation of CO_2_ was considered, the significant conversion of CO_2_ was only observed over the Ce-containing catalysts of PtSnCe/SiO_2_ and PtCe/SiO_2_, while CO_2_ conversion for PtSn/SiO_2_ was negligible. This indicates that the introduced CeO_2_ plays a key role in the activation of CO_2_, which is supported by our previous work for oxidative dehydrogenation of ethylbenzene with CO_2_ [53]. To shed some light on these observations, C_3_H_8_- and CO_2_-TPD experiments were performed over the catalysts. In the case of C_3_H_8_-TPD (Figure 7a), a very broad curve was observed for all of the catalysts in the temperature range of 100–400 °C, indicating the varied strength of adsorbed propane [54,55]. For PtSn/SiO_2_, two overlapping peaks were clearly observed at about 118 °C and 236 °C, respectively. When PtCe/SiO_2_ and PtSnCe/SiO_2_ were considered, the peak maxima were shifted toward higher temperatures in comparison with those of PtSn/SiO_2_. Moreover, the peak areas of desorbed propane were significantly increased. This indicates the intensified adsorption of propane over the Ce-containing catalysts. The amount of desorbed propane was calculated below 400 °C during C_3_H_8_-TPD, and the results are given in Table 3. It was increased in the order of PtSn/SiO_2_ << PtCe/SiO_2_ < PtSnCe/SiO_2_, which coincides well with the propane conversion. This clearly reveals that the amount of adsorbed propane plays a key role in determining the activity of these catalysts for CO_2_-ODP, which can be reasonably associated with Pt dispersion. As for the adsorption of CO_2_, a broad CO_2_-TPD pattern similar to that of C_3_H_8_-TPD was obtained for all of the catalysts (Figure 7b). For PtSn/SiO_2_, only a small peak was observed at about 126 °C, indicating the very weak adsorption of CO_2_, which is consistent with references [56,57]. In contrast, the peak temperature of desorbed CO_2_ increased to 141 °C over PtCe/SiO_2_ and PtSnCe/SiO_2_. Moreover, a shoulder peak could be observed at a higher temperature of 242 °C, which can be explained by the stronger adsorbed CO_2_ on the surface of CeO_2_. This indicates the presence of CeO_2_-enhanced CO_2_ adsorption. As shown in Table 3, the amount of desorbed CO_2_ was calculated below 400 °C during CO_2_-TPD. It was increased in the order of PtSn/SiO_2_ << PtCe/SiO_2_ < PtSnCe/SiO_2_, the changing pattern of which coincides well with that of CO_2_ conversion at the steady state of TOS (Figure 1b). It has been reported that CeO_2_ with richer oxygen defects commonly leads to enhanced adsorption and activation of CO_2_ [21,32]. Following this understanding, the greater amount of adsorbed CO_2_ in the case of PtSnCe/SiO_2_ than that of PtCe/SiO_2_ can be reasonably ascribed to the presence of more oxygen defects of CeO_2_, as revealed by the Ce 3d XPS results (Table 2). These results indicate that the amount of adsorbed CO_2_ plays a key role in determining the activation of CO_2_ over the catalysts, which can be connected with the introduced CeO_2_.

### 4.2. Insights into Product Selectivity

**Figure 8:**
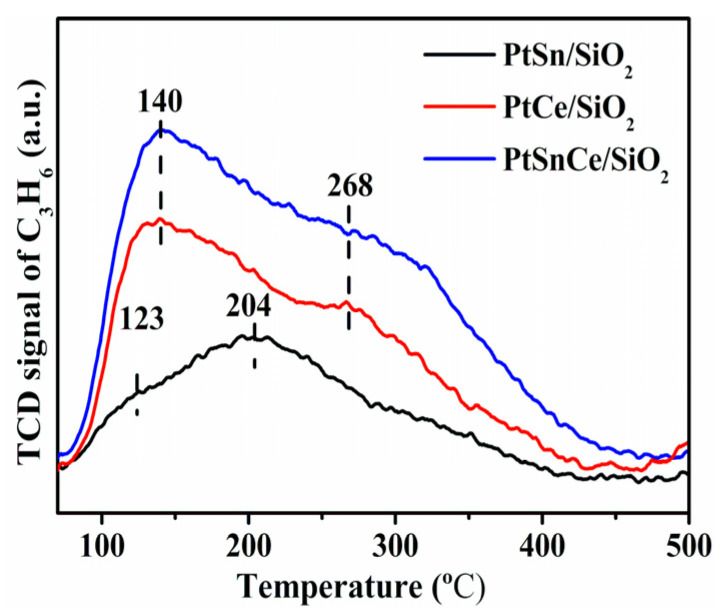
C_3_H_6_-TPD profiles of the reduced catalysts.

As indicated by the results in Section 3.1, the selectivity of propylene varied to a relatively large extent over the PtSn/SiO_2_, PtCe/SiO_2_, and PtSnCe/SiO_2_ catalysts (Figure 1c). According to the analysis of product distribution (Figure 1d), this is explained by the simultaneous occurrence of CO_2_-RP in the course of CO_2_-ODP. By correlating the characterization results of Section 3.2, Section 3.3, Section 3.4 and Section 3.5, the significant propylene selectivity over PtSnCe/SiO_2_ can be explained as the Ce promoted interaction between Sn and Pt, which favors the breaking of C-H bonds in propane [23,58]. However, in addition to the side reaction of CO_2_-RP induced from propane, the selectivity of propylene is also determined by its possible secondary reactions, including successive polymerization (coke deposition) and further cracking, owing to the difficult desorption of propylene from the surface of catalysts [59]. For further insight, C_3_H_6_-TPD experiments were performed, and the results are given in Figure 8. A very broad desorption signal was observed in the temperature range of 100 to 450 °C for all of the catalysts, indicating the varied strength of propylene adsorption on the surface of the catalyst [54]. In the case of PtSn/SiO_2_, two overlapping peaks were observed at about 123 °C and 204 °C. In contrast, both the peak temperature and amount of desorbed propylene over PtCe/SiO_2_ and PtSnCe/SiO_2_ were clearly higher than over PtSn/SiO_2_, indicating a stronger adsorption of propylene. Furthermore, as given in Table 3, the amount of adsorbed propylene was calculated below 450 °C during C_3_H_6_-TPD and was found to increase in the order of PtSn/SiO_2_ << PtCe/SiO_2_ < PtSnCe/SiO_2_. When propylene selectivity is compared with the amount of propylene adsorption, exactly the same trend is found, i.e., the greater the amount of propylene adsorption, the higher the propylene selectivity. This result is contradictory to the common expectation. Considering the dominant byproduct of CO in the course of CO_2_-ODP (Figure 1d), it can be concluded that the simultaneous occurrence of CO_2_-RP plays a key role in determining the propylene selectivity in comparison with the secondary reaction of propylene.

As a matter of fact, coke deposition on the surface of catalysts is a common issue in the course of CO_2_-ODP, the behavior of which is associated with its catalytic performance [4,54]. TG-DSC was performed to analyze the amount and kind of deposited coke over the spent catalysts after a TOS of 2 h, and the results are shown in Figure S6 and Table 3. For all of the catalysts, a clear weight loss was observed at about 30–200 °C, induced from the physical desorption of water (Figure S6a), accompanying the clearly endothermic peak of DSC curves at around 74 °C (Figure S6b). With a further increase in temperature from 200 to 800 °C, the TG signal commonly assigned to the burning of deposited coke was almost steady in the case of PtSn/SiO_2_, indicating a negligible amount of coke formed on the surface. This coincides well with the significantly low propane conversion (Figure 1a). Contrary to this, a weight loss of 1.71% and 2.53% was clearly observed over PtCe/SiO_2_ and PtSnCe/SiO_2_, respectively, at about 300–600 °C, ascribed to the burning of coke. This was further revealed by the exothermic peak of DSC curves. Moreover, the peak temperature of DSC for PtSnCe/SiO_2_ (400 °C) was clearly lower than that for PtCe/SiO_2_ (452 °C), suggesting a difference in the degree of graphitization of the deposited coke. To further confirm this, visible Raman characterization was performed. As given in Figure S7, typical Raman shifts were observed over PtCe/SiO_2_ and PtSnCe/SiO_2_ at 1340 and 1600 cm^−1^, assigned to the disordered (D band) and graphitic carbon (G band), respectively. To quantify the extent of graphitization of the deposited coke, the intensity ratio of the D and G bands, i.e., I_D_/I_G_, was calculated. As shown in Table 3, PtSnCe/SiO_2_ showed a higher value of I_D_/I_G_ (0.81) than PtCe/SiO_2_ (0.73), indicating a lesser extent of graphitization of coke species [32]. This is in agreement with the DSC results (Figure S6b). The difference in the species of deposited coke can be explained by the fact that PtCe/SiO_2_ is favorable to CO_2_-RP, while PtSnCe/SiO_2_ is promising for CO_2_-ODP. The lesser extent of graphitization of coke species on the surface of PtSnCe/SiO_2_ mainly originated from the polymerization of the produced C_3_H_6_. However, PtCe/SiO_2_ led to the formation of more graphitic carbon species due to the severe breaking of the C-C bond in propane through CO_2_-RP. The coking rate (g/mol) of PtCe/SiO_2_ and PtSnCe/SiO_2_, defined as grams of deposited coke, was calculated following references [54,60] by converting 1 mole of propane after a TOS of 2 h. In the case of PtSnCe/SiO_2_, the coking rate was 0.07 g/mol, which is clearly lower than that of PtCe/SiO_2_ (2.09 g/mol). This indicates that coke deposition over PtSnCe/SiO_2_ is significantly inhibited, which may result from the lesser extent of graphitization of coke species for CO_2_-ODP.

## 5. Conclusions

In summary, a highly efficient CO_2_-ODP catalyst was developed with STY_C3H6_ as high as 1.75 g(C_3_H_6_)·g(catalyst)^−^·h^−1^ by simply impregnating Ce (6 wt%) into PtSn/SiO_2_. Moreover, CO_2_-ODP performance was essentially restored after the regeneration of the catalyst at 500 °C for 30 min in an air flow. Additionally, the promotional effect of CeO_2_ on PtSn/SiO_2_ played a key role in determining the initial CO_2_-ODP performance, leading to the same increased order of PtSn/SiO_2_ < PtCe/SiO_2_ < PtSnCe/SiO_2_ for the initial propane conversion of 4.4%, 20.6%, and 55.8% and propylene selectivity of 31.0%, 39.7%, and 89.1%. Physical, chemical, and spectra characterizations reveal that the addition of CeO_2_ led to an increased Pt dispersion of 13.4% for PtSn/SiO_2_ < 20.9% for PtCe/SiO_2_ < 41.3% for PtSnCe/SiO_2_ and strong interactions between Pt and Sn species over the PtSnCe/SiO_2_ catalyst, which favors the synchronized activation of C-H bonds in propane and the C=O bonds in CO_2_ molecules. This was explained as the enhanced adsorption of propane and CO_2_ in the order of PtSn/SiO_2_ < PtCe/SiO_2_ < PtSnCe/SiO_2_, essentially originated from the rich oxygen defects over the added CeO_2_. With these understandings, the modification of catalysts with improved oxygen defects over oxides, as well as the search for promoters with richer oxygen defects than CeO_2_, is expected to produce a more effective Pt-based catalyst for CO_2_-ODP, with additional studies still in progress in our laboratory.

## Figures and Tables

**Table 1:** Textural properties, crystallite size, and metal dispersion of the different catalysts.

Catalyst	Specific Surface Area (m^2^/g)	Mean Pore Size (nm)	Total Pore Volume (cm^3^/g)	Crystallite Size (nm)		Metal Dispersion *** (%)
				Pt *	CeO_2_ **	
PtSn/SiO_2_	568.9	3.19	0.42	8.5 ± 0.2	-	13.4
PtCe/SiO_2_	536.4	3.25	0.43	3.4 ± 0.2	5.9	20.9
PtSnCe/SiO_2_	527.3	3.16	0.41	2.1 ± 0.3	5.6	41.3

*: The average size of 100 particles randomly selected in the TEM images was measured using Nano Measurer 1.2.5 software. **: calculated from Scherrer’s equation and the (111) diffraction of the cubic CeO_2_, as given in the XRD patterns. ***: calculated from Equation (1) in Section 2.2 and the H_2_-O_2_ titration results.

**Table 2:** H_2_ consumption during H_2_-TPR below 600 °C and XPS peak-fitting results of different catalysts.

Catalyst	H_2_ Consumption (mol/g)	Pt^0^ (%)	Pt^2+^ (%)	Sn^0^ (%)	Ce^3+^ (%)
PtSn/SiO_2_	23.5	100.0	0	0	-
PtCe/SiO_2_	78.5	30.5	69.5	-	31.9
PtSnCe/SiO_2_	140.6	33.1	66.9	12.0	43.2

**Table 3:** Calculated results of C_3_H_8_-, CO_2_-, and C_3_H_6_-TPD and the coking property of different catalysts.

Catalyst	C_3_H_8_	CO_2_	C_3_H_6_	Coke Content (wt%)	I_D_/I_G_	Coking Rate (g/mol)
	(mmol/g) *					
PtSn/SiO_2_	0.09	0.04	0.10	-	-	-
PtCe/SiO_2_	0.22	0.11	0. 23	1.71	0.73	2.09
PtSnCe/SiO_2_	0.30	0.14	0.26	2.53	0.81	0.07

*: Amounts of desorbed C_3_H_8_, CO_2_, and C_3_H_6_ were determined from the TPD patterns given in Figure 6 and Figure 7.

## Data Availability

The data presented in this study are available on request from the corresponding author.

## Supplementary Materials

The following supporting information can be downloaded at: https://www.mdpi.com/article/10.3390/nano12030417/s1. Figure S1: Carbon balances of CO_2_-ODP over the PtSn/SiO_2_, PtCe/SiO_2_, and PtSnCe/SiO_2_ catalysts at different times on stream, Figure S2: Time-on-stream catalytic activity for CO_2_-ODP over SnCe/SiO_2_, Figure S3: Time-on-stream selectivity of CH_4_, C_2_H_4_, and C_2_H_6_, Figure S4: N_2_ adsorption/desorption isotherms and pore-size distributions determined by the BJH method for PtSn/SiO_2_, PtCe/SiO_2_ and PtSnCe/SiO_2_ catalysts, Figure S5: Ce 3d XPS spectra of the reduced PtCe/SiO_2_ and PtSnCe/SiO_2_ catalysts, Figure S6: TG and DSC patterns of the catalysts after CO_2_-ODP for a time on stream of 2 h, Figure S7: Raman spectra of the catalysts after CO_2_-ODP for a time on stream of 2 h, Table S1: List of the reaction conditions and the main results of CO_2_-ODP over different catalysts [13,16,54,61,62,63,64,65,66,67,68].

## References

[1] Amghizar I., Vandewalle L.A., Van K.M., Marin G.B. (2017). New trends in olefin production. Engineering.

[2] Galvis H.M.T., Jong K.P. (2013). Catalysts for production of lower olefins from synthesis gas: A review. ACS Catal..

[3] Sattler J.J., Ruiz-Martinez J., Santillan-Jimenez E., Weckhuysen B.M. (2014). Catalytic dehydrogenation of light alkanes on metals and metal oxides. Chem. Rev..

[4] Atanga M.A., Rezaei F., Jawad A., Fitch M., Rownaghi A.A. (2018). Oxidative dehydrogenation of propane to propylene with carbon dioxide. Appl. Catal. B.

[5] Zhang L., Wang Z.-Y., Song J., Lang Y., Chen J.-G., Luo Q.-X., He Z.-H., Wang K., Liu Z.-W., Liu Z.-T. (2020). Facile synthesis of SiO_2_ supported GaN as an active catalyst for CO_2_ enhanced dehydrogenation of propane. J. CO_2_ Util..

[6] Jiang X., Sharma L., Fung V., Park S.J., Jones C.W., Sumpter B.G., Baltrusaitis J., Wu Z. (2021). Oxidative dehydrogenation of propane to propylene with soft oxidants via heterogeneous catalysis. ACS Catal..

[7] Chen S., Zeng L., Mu R., Xiong C., Zhao Z.J., Zhao C., Pei C., Peng L., Luo J., Fan L.S. (2019). Modulating lattice oxygen in dual-functional Mo-V-O mixed oxides for chemical looping oxidative dehydrogenation. J. Am. Chem. Soc..

[8] Li G., Liu C., Cui X., Yang Y., Shi F. (2021). Oxidative dehydrogenation of light alkanes with carbon dioxide. Green Chem..

[9] Carrero C.A., Schloegl R., Wachs I.E., Schomaecker R. (2014). Critical literature review of the kinetics for the oxidative dehydrogenation of propane over well-defined supported vanadium oxide catalysts. ACS Catal..

[10] Wang S., Zhu Z.H. (2004). Catalytic conversion of alkanes to olefins by carbon dioxide oxidative dehydrogenations—A review. Energy Fuel.

[11] Wegrzyniak A., Jarczewski S., Wegrzynowicz A., Michorczyk B., Kustrowski P., Michorczyk P. (2017). Catalytic behavior of chromium oxide supported on nanocasting-prepared mesoporous alumina in dehydrogenation of propane. Nanomaterials.

[12] Ruthwik N., Kavya D., Shadab A., Lingaiah N., Sumana C. (2020). Thermodynamic analysis of chemical looping combustion integrated oxidative dehydrogenation of propane to propylene with CO_2_. Chem. Eng. Process..

[13] Gomez E., Kattel S., Yan B., Yao S., Liu P., Chen J.G. (2018). Combining CO_2_ reduction with propane oxidative dehydrogenation over bimetallic catalysts. Nat. Commun..

[14] Ascoop I., Galvita V.V., Alexopoulos K., Reyniers M.-F., Voort P.V.D., Bliznuk V., Marin G.B. (2016). The role of CO_2_ in the dehydrogenation of propane over WO_x_-VO_x_/SiO_2_. J. Catal..

[15] Shishido T., Shimamura K., Teramura K., Tanaka T. (2012). Role of CO_2_ in dehydrogenation of propane over Cr-based catalysts. Catal. Today.

[16] Nowicka E., Reece C., Althahban S.M., Mohammed K.M.H., Kondrat S.A., Morgan D.J., He Q., Willock D.J., Golunski S., Kiely C.J. (2018). Elucidating the role of CO_2_ in the soft oxidative dehydrogenation of propane over ceria-based catalysts. ACS Catal..

[17] Dai Y., Gao X., Wang Q., Wan X., Zhou C., Yang Y. (2021). Recent progress in heterogeneous metal and metal oxide catalysts for direct dehydrogenation of ethane and propane. Chem. Rev..

[18] Zhang J., Deng Y., Cai X., Chen Y., Peng M., Jia Z., Jiang Z., Ren P., Yao S., Xie J. (2019). Tin-assisted fully exposed platinum clusters stabilized on defect-rich graphene for dehydrogenation reaction. ACS Catal..

[19] Kumar M.S., Chen D., Holmen A., Walmsley J.C. (2009). Dehydrogenation of propane over Pt-SBA-15 and Pt-Sn-SBA-15: Effect of Sn on the dispersion of Pt and catalytic behavior. Catal. Today.

[20] Chen S., Chang X., Sun G., Zhang T., Xu Y., Wang Y., Pei C., Gong J. (2021). Propane dehydrogenation: Catalyst development, new chemistry, and emerging technologies. Chem. Rev..

[21] Etim U.J., Zhang C., Zhong Z. (2021). Impacts of the catalyst structures on CO_2_ activation on catalyst surfaces. Nanomaterials.

[22] Wang H., Yang G.-Q., Song Y.-H., Liu Z.-T., Liu Z.-W. (2019). Defect-rich Ce_1-x_Zr_x_O_2_ solid solutions for oxidative dehydrogenation of ethylbenzene with CO_2_. Catal. Today.

[23] Xue M., Zhou Y., Zhang Y., Liu X., Duan Y., Sheng X. (2012). Effect of cerium addition on catalytic performance of PtSnNa/ZSM-5 catalyst for propane dehydrogenation. J. Nat. Gas Chem..

[24] Motagamwala A.H., Almallahi R., Wortman J., Igenegbai V.O., Linic S. (2021). Stable and selective catalysts for propane dehydrogenation operating at thermodynamic limit. Science.

[25] Sun Q., Wang N., Fan Q., Zeng L., Mayoral A., Miao S., Yang R., Jiang Z., Zhou W., Zhang J. (2020). Subnanometer bimetallic platinum-zinc clusters in zeolites for propane dehydrogenation. Angew. Chem. Int. Ed..

[26] Akhtar M., Tompkins F.C. (1971). The hydrogen-oxygen titration on platinum films: Determination of the catalytically active area. Trans. Faraday Soc..

[27] Wang T., Jiang F., Liu G., Zeng L., Zhao Z., Gong J. (2016). Effects of Ga doping on Pt/CeO_2_-Al_2_O_3_ catalysts for propane dehydrogenation. AIChE J..

[28] Lin C., Yang Z., Pan H., Cui J., Lv Z., Liu X., Tian P., Xiao Z., Li P., Xu J. (2021). Ce-introduced effects on modification of acidity and Pt electronic states on Pt-Sn/γ-Al_2_O_3_ catalysts for catalytic reforming. Appl. Catal. A Gen..

[29] Baek J., Yun H.J., Yun D., Choi Y., Yi J. (2012). Preparation of highly dispersed chromium oxide catalysts supported on mesoporous silica for the oxidative dehydrogenation of propane using CO_2_: Insight into the nature of catalytically active chromium sites. ACS Catal..

[30] Deng L., Miura H., Ohkubo T., Shishido T., Wang Z., Hosokawa S., Teramura K., Tanaka T. (2019). The importance of direct reduction in the synthesis of highly active Pt-Sn/SBA-15 for n-butane dehydrogenation. Catal. Sci. Technol..

[31] Kaneko S., Izuka M., Takahashi A., Ohshima M., Kurokawa H., Miura H. (2012). Pt dispersion control in Pt/SiO_2_ by calcination temperature using chloroplatinic acid as catalyst precursor. Appl. Catal. A Gen..

[32] Wang H., Cao F.-X., Song Y.-H., Yang G.-Q., Ge H.-Q., Liu Z.-T., Qu Y.-Q., Liu Z.-W. (2019). Two-step hydrothermally synthesized Ce_1-x_Zr_x_O_2_ for oxidative dehydrogenation of ethylbenzene with carbon dioxide. J. CO_2_ Util..

[33] Deng L., Miura H., Shishido T., Wang Z., Hosokawa S., Teramura K., Tanaka T. (2018). Elucidating strong metal-support interactions in Pt-Sn/SiO_2_ catalyst and its consequences for dehydrogenation of lower alkanes. J. Catal..

[34] Huang L., Xu B., Yang L., Fan Y. (2008). Propane dehydrogenation over the PtSn catalyst supported on alumina-modified SBA-15. Catal. Commun..

[35] Wang H., Huang H., Bashir K., Li C. (2020). Isolated Sn on mesoporous silica as a highly stable and selective catalyst for the propane dehydrogenation. Appl. Catal. A Gen..

[36] Chen A., Zhou Y., Ta N., Li Y., Shen W. (2015). Redox properties and catalytic performance of ceria-zirconia nanorods. Catal. Sci. Technol..

[37] Zhang H., Wang J., Zhang Y., Jiao Y., Ren C., Gong M., Chen Y. (2016). A study on H_2_ -TPR of Pt/Ce_0.27_Zr_0.73_O_2_ and Pt/Ce_0.27_Zr_0.70_ La_0.03_O_x_ for soot oxidation. Appl. Surf. Sci..

[38] Lee J., Ryou Y., Chan X., Kim T.J., Kim D.H. (2016). How Pt interacts with CeO_2_ under the reducing and oxidizing environments at elevated temperature: The origin of improved thermal stability of Pt/CeO_2_ compared to CeO_2_. J. Phys. Chem. C.

[39] Xiang X., Zhao H., Yang J., Zhao J., Yan L., Song H., Chou L. (2016). Nickel based mesoporous silica-ceria-zirconia composite for carbon dioxide reforming of methane. Appl. Catal. A Gen..

[40] Ono L.K., Croy J.R., Heinrich H., Roldan Cuenya B. (2011). Oxygen chemisorption, formation, and thermal stability of Pt oxides on Pt nanoparticles supported on SiO_2_/Si(001): Size effects. J. Phys. Chem. C.

[41] Bruix A., Lykhach Y., Matolínová I., Neitzel A., Skála T., Tsud N., Vorokhta M., Stetsovych V., Ševčíková K., Mysliveček J. (2014). Maximum noble-metal efficiency in catalytic materials: Atomically dispersed surface platinum. Angew. Chem. Int. Ed..

[42] Xiong H., Lin S., Goetze J., Pletcher P., Guo H., Kovarik L., Artyushkova K., Weckhuysen B.M., Datye A.K. (2017). Thermally stable and regenerable platinum-tin clusters for propane dehydrogenation prepared by atom trapping on ceria. Angew. Chem. Int. Ed..

[43] Zhu H., Anjum D.H., Wang Q., Abou-Hamad E., Emsley L., Dong H., Laveille P., Li L., Samal A.K., Basset J.-M. (2014). Sn surface-enriched Pt-Sn bimetallic nanoparticles as a selective and stable catalyst for propane dehydrogenation. J. Catal..

[44] Xu H., Wen C., Liu H., Li Z.P., Shen W.Z. (2013). Relationship of microstructure properties to oxygen impurities in nanocrystalline silicon photovoltaic materials. J. Appl. Phys..

[45] Balakrishnan K., Sachdev A., Schwank J. (1990). Chemisorption and FTIR study of bimetallic Pt-Au/SiO_2_ catalysts. J. Catal..

[46] Podda N., Corva M., Mohamed F., Feng Z., Dri C., Dvorák F., Matolin V., Comelli G., Peressi M., Vesselli E. (2016). Experimental and theoretical investigation of the restructuring process induced by CO at near ambient pressure: Pt nanoclusters on graphene/Ir(111). ACS Nano.

[47] Arteaga G.J., Anderson J.A., Rochester C.H. (1999). FTIR study of CO adsorption on coked Pt-Sn/Al_2_O_3_ catalysts. Catal. Lett..

[48] Wang H.-Z., Zhang W., Jiang J.-W., Sui Z.-J., Zhu Y.-A., Ye G.-H., Chen D., Zhou X.-G., Yuan W.-K. (2019). The role of H_2_S addition on Pt/Al_2_O_3_ catalyzed propane dehydrogenation: A mechanistic study. Catal. Sci. Technol..

[49] Boccuzzi F., Chiorino A., Guglielminotti E. (1996). Effects of structural defects and alloying on the FTIR spectra of CO adsorbed on PtZnO. Surf. Sci..

[50] Wang Q., Tichit D., Meunier F., Guesmi H. (2020). Combined DRIFTS and DFT study of CO adsorption and segregation modes in Pt-Sn nanoalloys. J. Phys. Chem. C.

[51] Moscu A., Schuurman Y., Veyre L., Thieuleux C., Meunier F. (2014). Direct evidence by in situ IR CO monitoring of the formation and the surface segregation of a Pt-Sn alloy. Chem. Commun..

[52] Zhang W., Wang H., Jiang J., Sui Z., Zhu Y., Chen D., Zhou X. (2020). Size Dependence of Pt catalysts for propane dehydrogenation: From atomically dispersed to nanoparticles. ACS Catal..

[53] Wang H., Zhu W., Yang G.-Q., Zhang Y.-W., Song Y.-H., Jiang N., Liu Z.-T., Liu Z.-W. (2019). Insights into the oxidative dehydrogenation of ethylbenzene with CO_2_ catalyzed by the ordered mesoporous V_2_O_5_-Ce_0.5_Zr_0.5_O_2_-Al_2_O_3_. Ind. Eng. Chem. Res..

[54] Wang J., Song Y.-H., Liu Z.-T., Liu Z.-W. (2021). Active and selective nature of supported CrO_x_ for the oxidative dehydrogenation of propane with carbon dioxide. Appl. Catal. B.

[55] Hu Z.-P., Wang Y., Yang D., Yuan Z.-Y. (2020). CrOx supported on high-silica HZSM-5 for propane dehydrogenation. J. Energy Chem..

[56] Burri D.R., Choi K.M., Han D.S., Jiang N., Burri A., Park S.E. (2008). Oxidative dehydrogenation of ethylbenzene to styrene with CO_2_ over SnO_2_-ZrO_2_ mixed oxide nanocomposite catalysts. Catal. Today.

[57] Choi H., Oh S., Park J.Y. (2020). High methane selective Pt cluster catalyst supported on Ga_2_O_3_ for CO_2_ hydrogenation. Catal. Today.

[58] Yu C., Ge Q., Xu H., Li W. (2006). Effects of Ce addition on the Pt-Sn/γ-Al_2_O_3_ catalyst for propane dehydrogenation to propylene. Appl. Catal. A Gen..

[59] Shi L., Deng G.M., Li W.C., Miao S., Wang Q.N., Zhang W.P., Lu A.H. (2015). Al_2_O_3_ nanosheets rich in pentacoordinate Al^3+^ ions stabilize Pt-Sn clusters for propane dehydrogenation. Angew. Chem. Int. Ed..

[60] Yang G.-Q., He Y.-J., Song Y.-H., Wang J., Liu Z.-T., Liu Z.-W. (2021). Oxidative dehydrogenation of propane with carbon dioxide catalyzed by Zn_x_Zr_1-x_O_2-x_ solid solutions. Ind. Eng. Chem. Res..

[61] Wang H.-M., Chen Y., Yan X., Lang W.-Z., Guo Y.-J. (2019). Cr doped mesoporous silica spheres for propane dehydrogenation in the presence of CO_2_: Effect of Cr adding time in sol-gel process. Microporous and Mesoporous Mater..

[62] Michorczyk P., Ogonowski J., Zeńczak K. (2011). Activity of chromium oxide deposited on different silica supports in the dehydrogenation of propane with CO_2_—A comparative study. J. Mol. Catal. A Chem..

[63] Zhang F., Wu R., Yue Y., Yang W., Gu S., Miao C., Hua W., Gao Z. (2011). Chromium oxide supported on ZSM-5 as a novel efficient catalyst for dehydrogenation of propane with CO_2_. Microporous and Mesoporous Mater..

[64] Xue X.-L., Lang W.-Z., Yan X., Guo Y.J. (2017). Dispersed vanadium in three-dimensional dendritic mesoporous silica nanospheres: Active and stable catalysts for the oxidative dehydrogenation of propane in the presence of CO_2_. ACS Appl. Mater. Interfaces.

[65] Wang Z.-Y., He Z.-H., Xia Y., Zhang L., Wang K., Wang W., Yang Y., Chen J.-G., Liu Z.-T. (2021). Oxidative dehydrogenation of propane to propylene in the presence of CO_2_ over gallium nitride supported on NaZSM-5. Ind. Eng. Chem. Res..

[66] Xiao H., Zhang J., Wang P., Wang X., Pang F., Zhang Z., Tan Y. (2016). Dehydrogenation of propane over a hydrothermal-synthesized Ga_2_O_3_-Al_2_O_3_ catalyst in the presence of carbon dioxide. Catal. Sci. Technol..

[67] Chen M., Xu J., Cao Y., He H.-Y., Fan K.-N., Zhuang J.-H. (2010). Dehydrogenation of propane over In_2_O_3_-Al_2_O_3_ mixed oxide in the presence of carbon dioxide. J. Catal..

[68] Ren Y., Zhang F., Hua W., Yue Y., Gao Z. (2009). ZnO supported on high silica HZSM-5 as new catalysts for dehydrogenation of propane to propene in the presence of CO_2_. Catal. Today.

