# Supplementary material for: CeO2-Promoted PtSn/SiO2 as a High-Performance Catalyst for the Oxidative Dehydrogenation of Propane with Carbon Dioxide
Source: Nanomaterials (Basel). 2022 Jan 27;12(3):417. doi: 10.3390/nano12030417 (PMC8838316; doi:10.3390/nano12030417)
Supplement: Supplementary file 1 [file nanomaterials-12-00417-s001.zip › nanomaterials-1549014-supplementary.pdf]

Supplementary material

CeO_2-Promoted PtSn/SiO_2 as a High-Performance Catalyst for the Oxidative Dehydrogenation of Propane with Carbon Dioxide

Li Wang; Guo-Qing Yang; Xing Ren; Zhong-Wen Liu*

Key Laboratory of Syngas Conversion of Shaanxi Province, School of Chemistry & Chemical Engineering, Shaanxi Normal University, Xi'an 710119, China.

***: Corresponding author** (Z.-W. Liu)

No. 620, West Chang'an Avenue

School of Chemistry & Chemical Engineering

Shaanxi Normal University

Xi'an 710119, China

E-mail: zwliu@snnu.edu.cn

Table of Contents

1. Carbon balances of CO_2-ODP 3

2. Time-on-stream catalytic activity of SnCe/SiO_2 4

3. Time-on-stream selectivity of different by-products 5

4. N_2 adsorption/desorption isotherms of different catalysts 6

5. Ce 3d XPS spectra 7

6. TG-DSC results of spent catalysts 8

7. Raman results of spent catalysts 9

8. Comparison of different oxide and metal catalysts for CO_2-ODP 10

9. References 11

1. Carbon balances of CO_2-ODP

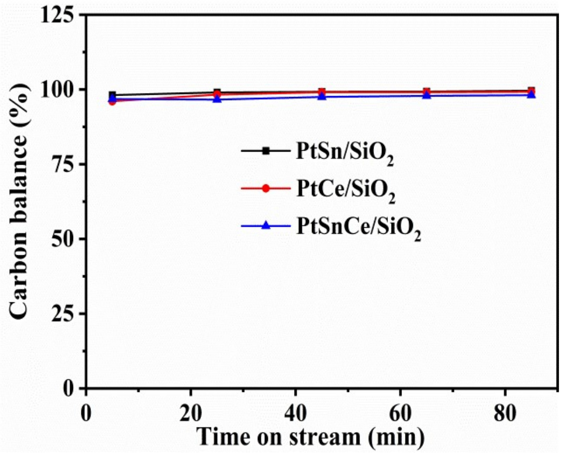

Figure S1. Carbon balances of CO_2-ODP over the PtSn/SiO_2, PtCe/SiO_2, and PtSnCe/SiO_2 catalyst at different time on streams (The reaction conditions are the same as those given in Figure 1).

2. Time-on-stream catalytic activity of SnCe/SiO_2

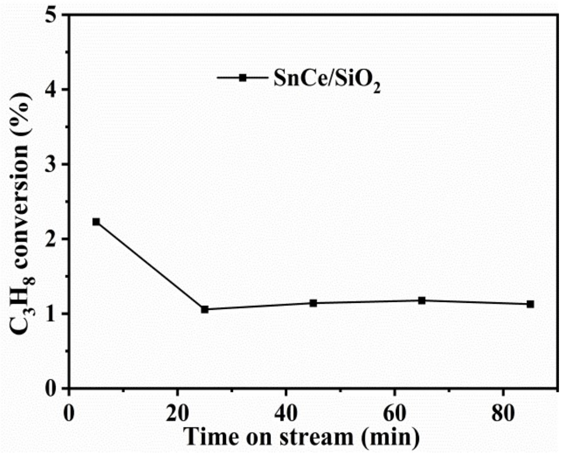

Figure S2. Time-on-stream catalytic activity for CO_2-ODP over SnCe/SiO_2 (The reaction conditions are the same as those given in Figure 1).

3. Time-on-stream selectivity of different by-products

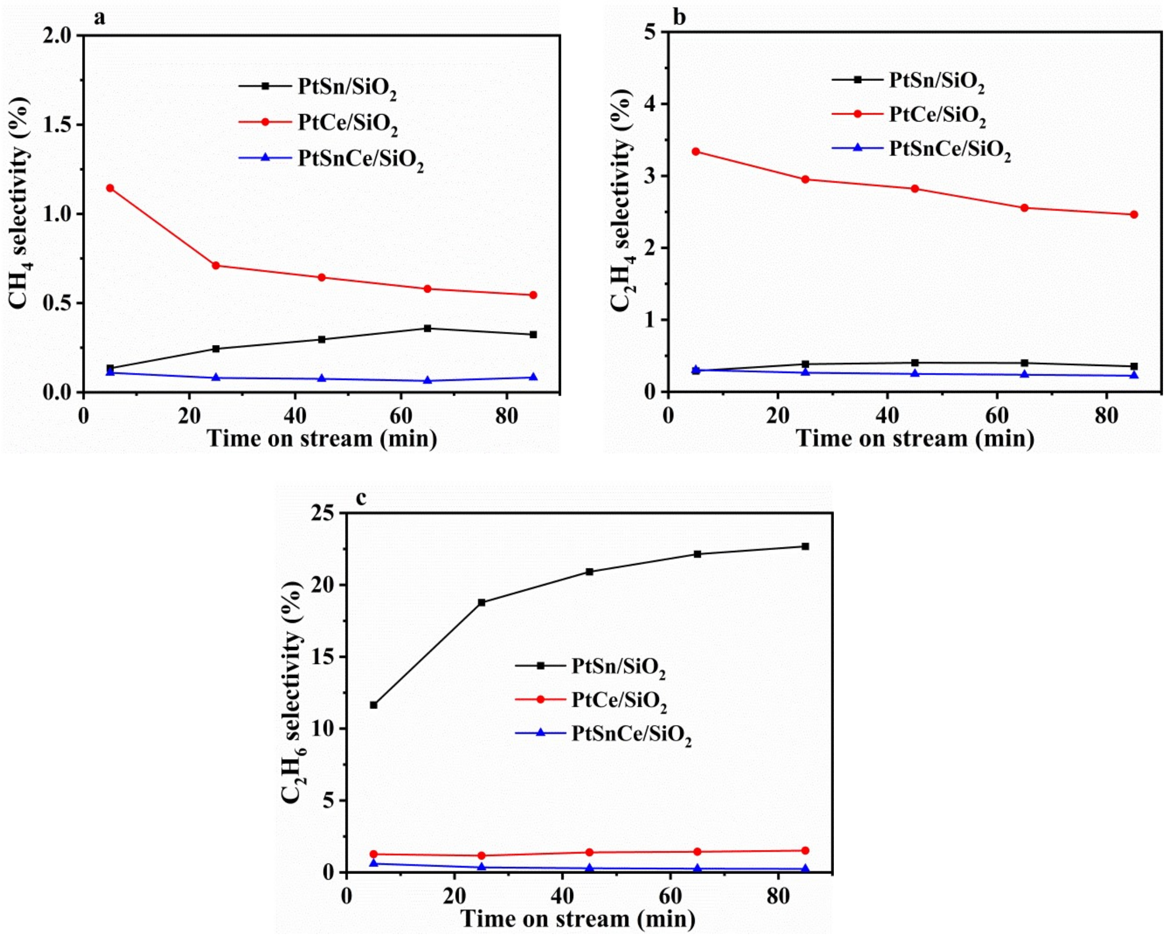

Figure S3. Time-on-stream selectivities of CH_4 (a), C_2H_4 (b), and C_2H_6 (c) (The reaction conditions are the same as those given in Figure 1).

4. N_2 adsorption/desorption isotherms of different catalysts

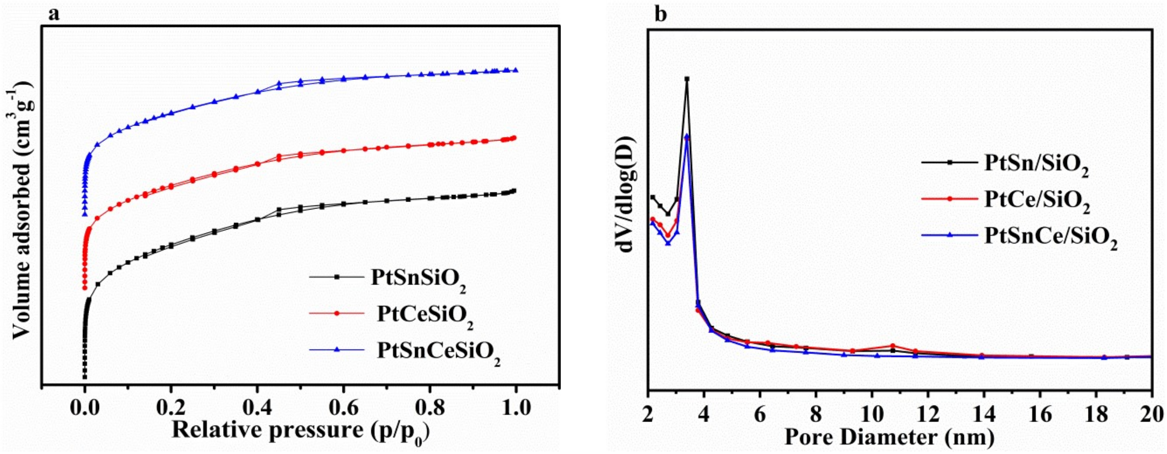

Figure S4. N_2 adsorption/desorption isotherms (a) and pore size distributions determined by the BJH method (b) for the PtSn/SiO_2, PtCe/SiO_2 and PtSnCe/SiO_2 catalyst.

5. Ce 3d XPS spectra

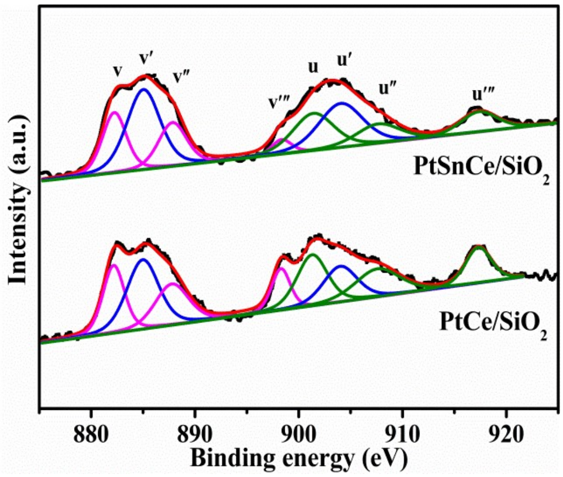

Figure S5. Ce 3d XPS spectra of the reduced PtCe/SiO_2 and PtSnCe/SiO_2 catalyst.

6. TG-DSC results of spent catalysts

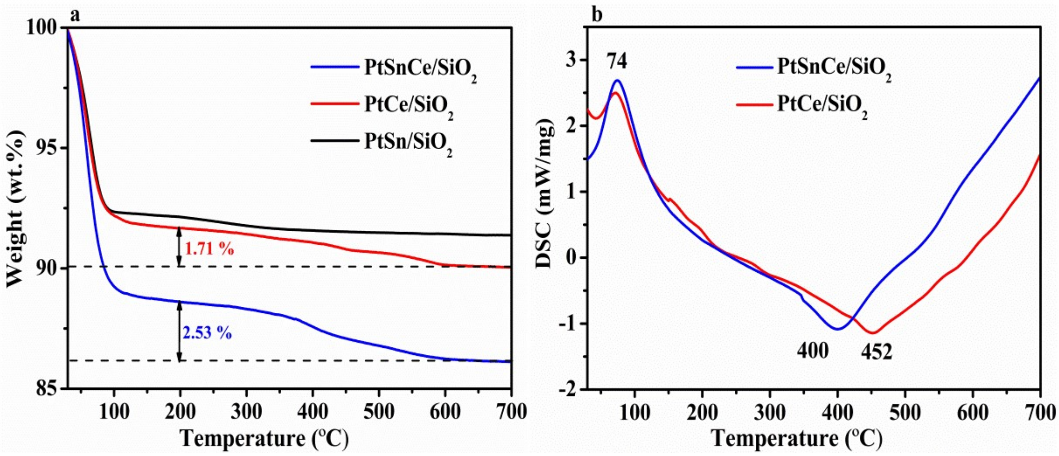

Figure S6. TG (a) and DSC (b) patterns of the catalysts after CO_2-ODP for a time on stream of 2 h (The reaction conditions are the same as those given in Figure 1).

7. Raman results of spent catalysts

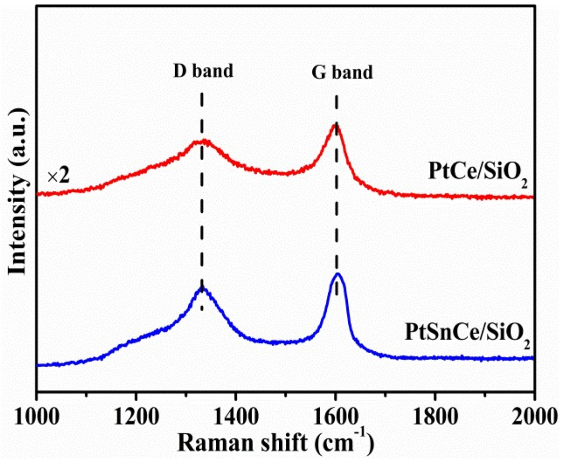

Figure S7. Raman spectra of the catalysts after CO_2-ODP for a time on stream of 2 h (The reaction conditions are the same as those given in Figure 1).

8. Comparison of different oxide and metal catalysts for CO_2-ODP

Table S1 List of the reaction conditions and the main results of CO_2-ODP over different catalysts

Catalyst	Reaction conditions					C_3H_6 yield (%)		Space time yield [g(C_3H_6)·g(catalyst)$^{-1}$·h^{-1}]		Reference
	Catalyst loadings (g)	Feed gas composition	T (ºC)	Total flow (ml/min)	TOS (h)	Initial	End	Initial	End	
7.07Cr/MSS-2	0.2	$2C_3H_8$+$8CO_2$+8Ar	600	18	3.3	55.7	29.0	0.63	0.33	[1]
Cr3.4/SBA-1	0.2	$1C_3H_8$+$5CO_2$+9He	550	30	7.7	29.2	18.1	0.29	0.18	[2]
3Cr/ZSM-5-S	0.2	$2.5C_3H_8$+$5CO_2$+$92.5N_2$	550	20	8.0	41.5	27.6	0.12	0.08	[3]
$2CrO_x$/silicalite-1	0.5	$4C_3H_8$+$20CO_2$+1He	550	25	2.1	34.6	29.2	0.33	0.28	[4]
5.2V-MSNSs	0.2	$1C_3H_8$+$4CO_2$+4Ar	600	15	2.0	47.9	31.0	0.45	0.29	[5]
5GaN/NaZSM-5(470)	0.2	$1C_3H_8$+$2CO_2$+$7N_2$	600	30	9.5	22.3	21.1	0.26	0.24	[6]
Ga_2O_3-Al_2O_3-HS	0.15	$1C_3H_8$+$3CO_2$+$27N_2$	550	15	9.0	23.5	8.1	0.06	0.02	[7]
In_2O_3-Al_2O_3-20	0.2	$1C_3H_8$+$4CO_2$+$35N_2$	600	10	12.0	17.1	13.5	0.02	0.02	[8]
ZnO/HZSM-5(160)	0.2	$1C_3H_8$+$2CO_2$+$37N_2$	600	20	30.0	31.9	25.8	0.09	0.07	[9]
5Pd/CeZrAlO$_x$	0.2	$37C_3H_8$+$37CO_2$+26He	500	15	142.6	13.0	2.8	0.41	0.09	[10]
Fe_3Ni/CeO_2	0.1	$1C_3H_8$+$1CO_2$+2Ar	550	40	13.3	1.6	1.5	0.18	0.16	[11]
Fe_3Pt/CeO_2	0.1	$1C_3H_8$+$1CO_2$+2Ar	550	40	13.3	1.7	0.4	0.19	0.04	[11]
**0.5%PtSnCe/SiO_2**	**0.25**	**$1C_3H_8$+$1CO_2$+5He**	**550**	**50**	**6.0**	**48.6**	**31.1**	**1.75**	**1.16**	**This work**

9. References

1. Wang, H.-M.; Chen, Y.; Yan, X.; Lang, W.-Z.; Guo, Y.-J. Cr doped mesoporous silica spheres for propane dehydrogenation in the presence of CO_2: effect of Cr adding time in sol-gel process. *Micropor. Mesopor. Mat.* **2019,** *284*, 69-77. [CrossRef]
2. Michorczyk, P.; Ogonowski, J.; Zeńczak, K. Activity of chromium oxide deposited on different silica supports in the dehydrogenation of propane with CO_2 - a comparative study. *J. Mol. Catal. A: Chem.* **2011,** *349*, 1-12. [CrossRef]
3. Zhang, F.; Wu, R.; Yue, Y.; Yang, W.; Gu, S.; Miao, C.; Hua, W.; Gao, Z. Chromium oxide supported on ZSM-5 as a novel efficient catalyst for dehydrogenation of propane with CO_2. *Micropor. Mesopor. Mat.* **2011,** *145*, 194-199. [CrossRef]
4. Wang, J.; Song, Y.-H.; Liu, Z.-T.; Liu, Z.-W. Active and selective nature of supported CrO_x for the oxidative dehydrogenation of propane with carbon dioxide. *Appl. Catal. B* **2021,** *297*, 120400. [CrossRef]
5. Xue, X. -L.; Lang, W. -Z.; Yan, X.; Guo, Y. J. Dispersed vanadium in three-dimensional dendritic mesoporous silica nanospheres: active and stable catalysts for the oxidative dehydrogenation of propane in the presence of CO_2. *ACS Appl. Mater. Inter.* **2017,** *9*, 15408-15423. [CrossRef]
6. Wang, Z. -Y.; He, Z. -H.; Xia, Y.; Zhang, L.; Wang, K.; Wang, W.; Yang, Y.; Chen, J.-G.; Liu, Z.-T. Oxidative dehydrogenation of propane to propylene in the presence of CO_2 over gallium nitride supported on NaZSM-5. *Ind. Eng. Chem. Res.* **2021,** *60*, 2807-2817. [CrossRef]
7. Xiao, H.; Zhang, J.; Wang, P.; Wang, X.; Pang, F.; Zhang, Z.; Tan, Y. Dehydrogenation of propane over a hydrothermal-synthesized Ga_2O_3-Al_2O_3 catalyst in the presence of carbon dioxide. *Catal. Sci. Technol.* **2016,** *6*, 5183-5195. [CrossRef]
8. Chen, M.; Xu, J.; Cao, Y.; He, H.-Y.; Fan, K.-N.; Zhuang, J.-H. Dehydrogenation of propane over In_2O_3-Al_2O_3 mixed oxide in the presence of carbon dioxide. *J. Catal.* **2010,** *272*, 101-108. [CrossRef]
9. Ren, Y.; Zhang, F.; Hua, W.; Yue, Y.; Gao, Z. ZnO supported on high silica HZSM-5 as new catalysts for dehydrogenation of propane to propene in the presence of CO_2. *Catal. Today* **2009,** *148*, 316-322. [CrossRef]
10. Nowicka, E.; Reece, C.; Althahban, S. M.; Mohammed, K. M. H.; Kondrat, S. A.; Morgan, D. J.; He, Q.; Willock, D. J.; Golunski, S.; Kiely, C. J.; Hutchings, G. J. Elucidating the role of CO_2 in the soft oxidative dehydrogenation of propane over ceria-based catalysts. *ACS Catal.* **2018,** *8*, 3454-3468. [CrossRef]
11. Gomez, E.; Kattel, S.; Yan, B.; Yao, S.; Liu, P.; Chen, J. G. Combining CO_2 reduction with propane oxidative dehydrogenation over bimetallic catalysts. *Nat. Commun.* **2018,** *9*, 1-6. [CrossRef]
